# IT’S NOT OK TO FALL: CHALLENGES AND SUCCESSES OF A NURSING HOME FALL PREVENTION PROJECT

**DOI:** 10.1093/geroni/igz038.2833

**Published:** 2019-11-08

**Authors:** Diana L Sturdevant, Barbara Carlson, Teri Round

**Affiliations:** 1 University of Oklahoma, Fran and Earl Ziegler College of Nursing, Oklahoma City, Oklahoma, United States; 2 University of Oklahoma, Fran and Earl Ziegler College of Nursing, Yukon, Oklahoma, United States

## Abstract

This study pilot-tested a 12-week, comprehensive, resident-centered fall prevention program aimed to lower falls in nursing home residents in Oklahoma. Staff from 52 nursing homes received a training on evidence-based fall prevention strategies and fall-risk assessment. Content was present using motivational scenarios that encouraged situational problems solving. Rate of falls, including falls with major injury) were collected for 3 months before (roll-in), during (treatment), and following (sustainability). Nursing homes completing the project (n=29) showed significant decline in falls, including falls with major injury. Staff acceptance and project implementation varied across nursing homes and was related to organizational policies that did not adapt care based on residents needs on any given day. Adaptability, not just knowledge, is important for ensuring the safety of residents. Future work will focus on the role of leadership in promoting more open systems for delivering care.

